# Accounting for Heterogeneity in Relative Treatment Effects for Use in Cost-Effectiveness Models and Value-of-Information Analyses

**DOI:** 10.1177/0272989X15570113

**Published:** 2015-07

**Authors:** Nicky J. Welton, Marta O. Soares, Stephen Palmer, Anthony E. Ades, David Harrison, Manu Shankar-Hari, Kathy M. Rowan

**Affiliations:** School of Social and Community Medicine, University of Bristol, Bristol, UK (NJW, AEA); Centre for Health Economics, University of York, York, UK (MOS, SP); Intensive Care National Audit & Research Centre, London, UK (DH, KMR); Guy’s and St Thomas’ Hospital NHS Foundation Trust, London, UK (MS-H)

**Keywords:** cost-effectiveness analysis, Bayesian meta-analysis, value of information

## Abstract

Cost-effectiveness analysis (CEA) models are routinely used to inform health care policy. Key model inputs include relative effectiveness of competing treatments, typically informed by meta-analysis. Heterogeneity is ubiquitous in meta-analysis, and random effects models are usually used when there is variability in effects across studies. In the absence of observed treatment effect modifiers, various summaries from the random effects distribution (random effects mean, predictive distribution, random effects distribution, or study-specific estimate [shrunken or independent of other studies]) can be used depending on the relationship between the setting for the decision (population characteristics, treatment definitions, and other contextual factors) and the included studies. If covariates have been measured that could potentially explain the heterogeneity, then these can be included in a meta-regression model. We describe how covariates can be included in a network meta-analysis model and how the output from such an analysis can be used in a CEA model. We outline a model selection procedure to help choose between competing models and stress the importance of clinical input. We illustrate the approach with a health technology assessment of intravenous immunoglobulin for the management of adult patients with severe sepsis in an intensive care setting, which exemplifies how risk of bias information can be incorporated into CEA models. We show that the results of the CEA and value-of-information analyses are sensitive to the model and highlight the importance of sensitivity analyses when conducting CEA in the presence of heterogeneity. The methods presented extend naturally to heterogeneity in other model inputs, such as baseline risk.

Cost-effectiveness analysis (CEA) models are routinely used to inform health care policy decisions between health care options.^1^ Relative treatment effects for effectiveness outcomes are among the key input parameters to CEA models. For policy decisions to reflect the evidence available, it is important that the relative effect inputs and their uncertainty are reflected appropriately in CEA models. CEA models are also used in value-of-information analyses that explore the need for, and optimal design of, new research studies.^2^

Relative treatment effects are typically obtained from meta-analyses or network meta-analyses (also termed *mixed treatment comparisons*)^3–5^ of all the available relevant randomized controlled trials (RCTs) that report the outcomes of interest. Ideally, all the RCTs estimate a common true treatment effect, and the only reason for differences between study estimates is sampling error. In this case, a fixed effect (FE) model can be used to deliver a single pooled estimate for each treatment comparison of interest. However, more often than not, there is additional variability between studies due to differences in inclusion criteria and/or trial conduct, such that the RCTs estimate different true treatment effects. It is then usually assumed that the study-specific true relative effects are *similar* in the sense that they can be described as coming from a common random effects (RE) distribution (usually assumed Normal).^6^ The relative effect that is reported is usually the mean of this distribution, although the predictive distribution or the entire RE distribution has been proposed,^7,8^ and some alternative approaches have been suggested recently in a short letter.^9^

Heterogeneity in relative effects can arise as a result of an imbalance in treatment effect–modifying patient characteristics across studies and/or in study-level parameters. If study-level parameters have been reported, then they can be included as covariates in a meta-regression model.^3,6,10,11^ Ideally, to avoid ecological bias,^12^ individual patient data are required to explore the impact of imbalance in patient-level characteristics, although for binary outcomes reported by subgroup, then aggregate-level data are sufficient, which is a simple example of meta-regression. Inclusions of treatment effect–modifying covariates are expected to lead to a reduction in heterogeneity and, in the extreme case, will eliminate heterogeneity entirely, reducing an RE model to an FE model. There may be many covariates that could potentially be included in a meta-regression, and a strategy is required to choose between models.

Where there is evidence of heterogeneity, careful thought needs to be given as to the most appropriate input to use in a CEA model. If subgroups have been identified, and it is acceptable to make different treatment recommendations in different subgroups, CEA models can be developed separately for each subgroup.^13^ The results from a CEA may be sensitive to the choice of which covariate model is selected, and so a structured approach to model selection is desirable. For example, an RE model with no covariates will result in less precise relative treatment effect estimates than those from an RE model in which some of the heterogeneity is explained by covariates. RE models can be summarized and interpreted in a variety of ways,^7–9^ and this needs to be reflected in a CEA model. Again, results from a CEA may be sensitive to this choice.^9^

In this article, we describe a general framework to model and account for heterogeneity in relative effect inputs to CEA models and set out a general strategy for model selection. Although we focus on relative treatment effects, the ideas extend naturally to other model inputs that exhibit heterogeneity. We illustrate the approaches with a recent health technology assessment of intravenous immunoglobulin (IVIG) for the management of adult patients with severe sepsis and septic shock.^14^ This case study exemplifies how risk of bias information can be incorporated into CEA models that, to our knowledge, has not been addressed in the literature previously.

## Methods

### Net Benefit

We assume there is a cost-effectiveness model with a net benefit function $\mathrm{NB}\left(k,d_{k}^{\mathrm{dec}},\theta \right)$ that depends on $d_{k}^{\mathrm{dec}}$, the relative effect of treatment k relative to treatment 1 in the target setting for the decision, and other input parameters θ, which includes treatment costs, natural history parameters such as baseline risk, resource use costs, adverse events, and utilities. By target “setting” for the decision, we include the patient population characteristics, treatment definitions, and other contextual factors. There may be uncertainty in all of the parameter inputs to the net benefit function, which need to be averaged over to obtain the expected net benefit, on which to base decisions. We assume that there is only a single effectiveness outcome relevant to the decision model.

### Meta-Analysis Models in the Absence of Covariates

In all that follows, we assume that relative effects are modeled on an appropriate scale (e.g., log-odds ratios for binary observations). When there are two treatments being compared, a pairwise FE meta-analysis model assumes that the true relative treatment effects, $\delta_{j}$, from study j are equal, $\delta_{j}=d_{2}$, where $d_{2}$ is the pooled mean effect of treatment 2 relative to treatment 1. For a pairwise RE meta-analysis model, the $\delta_{j}$ are assumed to come from a common distribution, for example, for a Normal RE model, $\delta_{j}\sim N(d_{2},\tau^{2})$, where $d_{2}$ is the mean of the RE distribution and $\tau^{2}$ is the between-study variance in treatment effects.

When there are several competing treatments, relative effect estimates can be obtained from a network meta-analysis^4^:

$$\begin{array}{cc} & \mathrm{Fixed Effect Model}:\;\delta_{j,k,b}=d_{k}-d_{b},\;d_{1}=0\\ & \mathrm{Random Effects Model}:\;\delta_{j,k,b}\sim N(d_{k}-d_{b},\tau^{2}),\;d_{1}=0 \end{array}$$

where $\delta_{j,k,b}$ is the estimated effect of treatment k in study j relative to the baseline (lowest numbered) treatment b for that study, and $d_{k}$ is the mean effect of treatment k relative to treatment 1. In the RE model, the between-study variance parameter is assumed the same for every treatment comparison (homogeneous variance assumption). If there are only two treatments, then equation (1) reduces to a standard pairwise meta-analysis.^4^

### Meta-Analysis Inputs to CEA in the Absence of Covariates

Note that in all that follows, parameter inputs are estimated with uncertainty, and this uncertainty needs to be averaged over to obtain expected net benefit.

For an FE model, assuming that the treatment effect in the target setting for the decision is no different from those in the studies that make up the (network) meta-analysis, then the $d_{k}^{\mathrm{dec}}$ are expected to be equal to the pooled treatment effect(s) from the (network) meta-analysis, so that the relevant effectiveness inputs to the CEA model and net benefit for the decision population are

$$d_{k}^{\mathrm{dec}}=d_{k}\;\mathrm{and}\;NB^{\mathrm{dec}}(k,d_{k},\theta )=\mathrm{NB}(k,d_{k},\theta ).$$

There are several possible approaches to summarize an RE model to provide inputs to a CEA model, which depend on our interpretation of the heterogeneity in the studies included in the (network) meta-analysis and how this relates to the target setting for the decision.^9^

(a) *Random effects mean*. The most commonly used approach is to use the mean of the RE distribution as the input for the CEA model, so that

$$d_{k}^{\mathrm{dec}}=d_{k}\;\mathrm{and}\;NB^{\mathrm{dec}}(k,d_{k},\theta )=\mathrm{NB}(k,d_{k},\theta ).$$

This assumes that the target setting for the decision is exactly equal to the average setting from the studies included in the (network) meta-analysis. This is unlikely to be the case in practice. One possible scenario when equation (3) might be appropriate is if the cause of the heterogeneity is due solely to bias resulting from flaws in study conduct but that the bias across studies is centered on 0. Then the RE mean represents the treatment effect in an unbiased study.

(b) *Predictive distribution*. Due to the difficulty in interpreting the RE mean, the predictive distribution has been proposed as a more realistic way to characterize the uncertainty in the treatment effect we may expect to see in the future. A prediction $d_{k}^{\mathrm{pred}}$ is drawn from the RE distribution, $d_{k}^{\mathrm{pred}}\sim N(d_{k},\tau^{2})$. $d_{k}^{\mathrm{pred}}$ has the same central estimate as the RE mean, $d_{k}$, but is less precise, because it reflects the uncertainty as to where a randomly selected study setting might lie in the RE distribution, as well as the uncertainty in the RE parameters $d_{k}$ and τ. The precision of $d_{k}^{\mathrm{pred}}$ therefore decreases with increasing heterogeneity. In this case:

$$d_{k}^{\mathrm{dec}}=d_{k}^{\mathrm{pred}}\;\mathrm{and}\;NB^{\mathrm{dec}}(k,d_{k}^{\mathrm{pred}},\theta )=\mathrm{NB}(k,d_{k}^{\mathrm{pred}},\theta ).$$

This assumes that the target setting for the decision is “similar” to those in the studies included in the (network) meta-analysis in the sense that $d_{k}^{\mathrm{dec}}$ comes from the same distribution of treatment effects, but we do not know where in the RE distribution $d_{k}^{\mathrm{dec}}$ lies. This may often be reasonable, but it may be the case that the target setting for the decision is more closely related to a subset or a single study in the (network) meta-analysis, in which case the predictive distribution will lead to biased and imprecise estimates.

(c) *Independent study-specific estimate*. If we consider the target setting for the decision to be represented by a single study population, $j^{\mathrm{dec}}$, and information obtained from all other study populations are irrelevant, then we use the effect estimate from that study alone (not from a meta-analysis) as the input to the CEA, so that

$$d_{k}^{\mathrm{dec}}=d_{j^{\mathrm{dec}},k}\;\mathrm{and}\;NB^{\mathrm{dec}}(k,d_{j^{\mathrm{dec}},k},\theta )=\mathrm{NB}(k,d_{j^{\mathrm{dec}},k},\theta ).$$

This might be the case if all studies except one are deemed to be at high risk of bias, and if this is the cause of the heterogeneity, then we may want to only use the results from the study not at high risk of bias. Note, however, that this approach is limited, as it can only be used for treatment comparisons that have been included in study $j^{\mathrm{dec}}$.

If a subset of the studies are considered representative, then either the pooled estimate from an FE model on that subset of studies or the predictive distribution from an RE model on that subset of studies may be used.

(d) *Shrunken study-specific estimate*. If we consider the target setting for the decision to be “similar to” those in the studies included in the (network) meta-analysis (as for the predictive distribution), but it is most closely represented by a single study population, $j^{\mathrm{dec}}$, then we may want to use the study-specific estimate for study $j^{\mathrm{dec}}$ estimated from an RE model, $\delta_{j^{\mathrm{dec}},k,1}$. This estimate, known as a shrunken estimate, will be drawn in toward the RE mean (depending on the relative size of the study and the degree of heterogeneity) and will be more precisely estimated than the study estimate alone, because it is “borrowing strength” from the other study estimates. Then the input to the CEA model is

$$d_{k}^{\mathrm{dec}}=\delta_{j^{\mathrm{dec}},k,1}NB^{\mathrm{dec}}(k,\delta_{j^{\mathrm{dec}},k,1},\theta )=\mathrm{NB}(k,\delta_{j^{\mathrm{dec}},k,1},\theta ).$$

If study $j^{\mathrm{dec}}$ does not include treatment *c*, we can apply the consistency equations (4), $d_{k,c}=d_{k}-d_{c}$, to obtain the study-specific shrunken effect of treatment *c* compared with treatment 1:

$$\delta_{j^{\mathrm{dec}},c,1}=\delta_{j^{\mathrm{dec}},k,b}-(d_{k}-d_{b})+d_{c}.$$

(e) *Random effects distribution*. If we consider the target setting for the decision to be made up of those included in the studies in the (network) meta-analysis, then we would expect heterogeneity estimated in the (network) meta-analysis to be also seen in the decision setting. This may be the case where there is inherent variation between clinicians delivering the treatments. It is then necessary to integrate over the entire RE distribution in the CEA model to obtain the net benefit for the decision population, $NB^{\mathrm{dec}}(k,d_{k},\tau ,\theta )$, which depends on the parameters of the RE distribution. For example, for a Normal RE model, $\delta_{k}\sim N(d_{k},\tau^{2})$, and

$$NB^{\mathrm{dec}}(k,d_{k},\tau ,\theta )=\int \mathrm{NB}\left(k,\delta_{k},\theta \right)\frac{1}{\tau \sqrt{2\pi }}e^{\overset{2}{-\left(\frac{\delta_{k}-d_{k}}{\tau }\right)}}d\delta_{k}.$$

As in all cases above, the uncertainty in the parameters $\left\{d_{k},\tau ,\theta \right\}$ must be averaged over to obtain the expected net benefit.

### Meta-Regression with Study-Reported Covariates

Allowing for covariates provides the potential to explain some of the heterogeneity. Covariates can include study characteristics, such as whether an in- or outpatient setting was taken, and patient characteristics, such as disease severity. Ideally, patient-level data would inform estimation of patient covariate effects, but in meta-analysis, it is often the case that only study-level summaries are available. Let $x_{i,j}$ be the observed value of covariate i reported in study j, and then the network meta-analysis model becomes^10,11^:

$$\begin{array}{cc} & \mathrm{Fixed Effect Model}:\;\\ \delta_{j,k,b}=(d_{k}+\sum_{i}\beta_{i,k}x_{i,j})-(d_{b}+\sum_{i}\beta_{i,b}x_{i,j}),\;d_{1}=0\\ & \mathrm{Random Effects Model}:\;\\ \delta_{j,k,b}\sim N((d_{k}+\sum_{i}\beta_{i,k}x_{i,j})-(d_{b}+\sum_{i}\beta_{i,b}x_{i,j}),\tau^{2}),\;d_{1}=0 \end{array}$$

where $\beta_{i,k}$ is the additional effect on treatment k compared with treatment 1, per unit change in covariate i and *d*_1_ = 0 (see above). If there are only two treatments, then equation (9) reduces to a standard pairwise meta-regression.^11^

### Meta-Analysis Inputs to CEA in the Presence of Covariates

The incorporation of covariates in an RE model may reduce the heterogeneity parameter or even reduce the RE model to an FE model in the extreme case. Equations (2) to (8) can all be applied to the resulting RE or FE model to obtain inputs to a CEA model. However, some adjustments are required to account for the covariates.

#### Binary covariates

Suppose covariate i is binary, for example, whether the intervention is given in an in- or outpatient setting. If the covariate is always present in the decision setting, then the adjusted estimates $\left(d_{k}+\beta_{i,k}\right)$ should replace $d_{k}$ in equations (2) to (8), whereas if always absent, equations (2) to (8) are unchanged. Suppose that we know from other data sources that the covariate is present *P*% of the time, then the net benefit defined in equations (2) to (8) needs to be averaged over the covariate distribution:

$$\frac{P}{100}*NB^{\mathrm{dec}}\left(k,d_{k}+\beta_{i,k},\theta \right)+\frac{100-P}{100}*NB^{\mathrm{dec}}\left(k,d_{k}+\beta_{i,k},\theta \right).$$

The uncertainty in the parameters $\left\{d_{k},\beta_{i,k},\theta \right\}$ must be averaged over to obtain the expected net benefit.

#### Continuous covariates

Suppose covariate i is continuous, for example, mean age or volume of operations undertaken per year. If the value of the covariate in the target setting for the decision is known (e.g., mean age is $x^{\mathrm{dec}}$), then we replace $d_{k}$ with $\left(d_{k}+\beta_{i,k}x^{\mathrm{dec}}\right)$ in equations (2) to (8). If the value of the covariate is expected to vary within the target setting for the decision—for example, the distribution of the volume of operations across hospitals may be known from national statistics to be $g(x^{\mathrm{dec}})$—then the net benefit defined in equations (2) to (8) is an average over the covariate distribution:

$$\int NB^{\mathrm{dec}}\left(k,d_{k}+\beta_{i,k}x^{\mathrm{dec}},\theta \right)g(x^{\mathrm{dec}})dx^{\mathrm{dec}}.$$

The same approach applies for study-level ordinal effect modifiers, but the integration is replaced by a sum over all possible levels of the covariate.

#### Multiple covariates

Where there are multiple covariates $x_{1},\ldots ,x_{I}$, the net benefit defined in equations (2) to (8) needs to be averaged over the *joint distribution* $g(x_{1}^{\mathrm{dec}},\ldots ,x_{I}^{\mathrm{dec}})$of those covariates in the decision setting. For continuous covariates, this is

$$\underset{x_{1}^{\mathrm{dec}},\ldots ,x_{I}^{\mathrm{dec}}}{\int \int \int }NB^{\mathrm{dec}}\left(k,d_{k}+\sum_{i=1}^{I}\beta_{i,k}x_{i}^{\mathrm{dec}},\theta \right)g(x_{1}^{\mathrm{dec}},\ldots ,x_{I}^{\mathrm{dec}}){\mathrm{dx}}_{1}^{\mathrm{dec}}\ldots {\mathrm{dx}}_{I}^{\mathrm{dec}}.$$

The integration is replaced by a summation for binary and ordinal covariates.

#### Heterogeneity in treatment definitions

Treatment definition is a common cause of heterogeneity (e.g., formulation, dose, timing, and duration of treatment). Where there is a clinical rationale for a differential effect across treatment definitions, each distinct definition should ideally be considered a separate treatment in a network meta-analysis (equation (1)). However, this approach may lead to an unconnected network of treatment comparisons, and even if it is connected, there may be only a limited amount of evidence on each comparison. An alternative is to include aspects of the treatment definition (e.g., dose) as covariates. This may be reasonable where there is good evidence on the functional dose-response relationship, with the potential to increase precision of effect estimates. However, the assumed relationship needs to be transparent and fit to the data assessed. Another alternative is to consider different groupings for the treatments, with fixed or random treatment effect within grouping,^10,15,16^ and compare model fit to help aid the choice of treatment definitions. For example, it could be that doses within a particular range are homogeneous but that very small or large doses lead to differential treatment effects.

#### Risk of bias and small study effects

There is some evidence that treatment effects are vulnerable to methodological flaws in study design that introduce a risk of bias.^17,18^ For example, if the randomization process in a trial is inadequately concealed, selection bias may be introduced. There is also evidence that treatment effects have a tendency to be stronger in smaller studies.^17,18^ Risk of bias indicators (e.g., whether randomization was adequately concealed or not) can be included in a meta-regression as binary or ordinal covariates, and the treatment estimate used to inform cost-effectiveness should be associated with studies at low risk of bias (i.e., having adjusted for risk of bias) in the CEA (i.e., set $x^{\mathrm{dec}}$= 0 and use $d_{k}$ in equations (2) to (8)).

Study size, $N_{j}$, can be treated as a continuous covariate, usually modeled as either $x_{i,j}=1/N_{j}$ or $x_{i,j}=1/\sqrt{N_{j}}$,^19,20^ so that an adjusted treatment effect can be obtained by setting $x_{i}^{\mathrm{dec}}=0$ (which corresponds to letting N→∞). If the relationship between treatment effect and sample size observed in the included studies can be assumed to continue as N→∞, then this will provide a bias-adjusted treatment effect estimate. However, the plausibility of the extrapolation beyond the largest observed study size should be considered.

### Model Selection Strategy

The choice of covariates to include may in part be driven by what is reported in the included studies but should also be supported by clinical experts. There may be several potential covariates for inclusion in the meta-regression, and a strategy is required to help choose between them. We propose the following approach to model selection (although note there may be other systematic approaches that can be taken):

Step 1 Fit FE and RE models with no covariates and use model fit statistics and the estimated heterogeneity parameter, τ, to choose between the models. If there is no evidence of heterogeneity, then there is no need to explore covariates. However, if there is evidence of heterogeneity, then proceed to step 2.
Step 2 Fit the FE model with each of the potential covariates alone (i.e., univariable models), including different network meta-analysis structures to capture heterogeneity in treatment definitions. Compare model fit statistics to identify the key covariates that explain some of the heterogeneity. For those key covariates, also fit an RE model. Note that models including covariates may fit equally well to an RE model without covariates, but τ will be lower if the covariate is explaining some heterogeneity. If the FE model with covariates fits as well as the RE model without covariates, then the covariates have explained the majority of the heterogeneity. Covariates for consideration should be guided by clinical input as well as what information is available.
Step 3 Consider combinations of the key covariates identified in step 2 by adding additional covariates and comparing model fit statistics and τ to identify which combinations of covariates best explain the heterogeneity.
Step 4 Report results from all of the best-fitting models that achieve a similar model fit, and obtain clinical input on the interpretation/justification of covariates to help guide model choice. If necessary, repeat the process in light of the clinical input.

If a Bayesian approach is taken, then the posterior mean residual deviance and deviance information criterion (DIC) measures^21^ for model fit and model comparison can be used. If a frequentist approach is used, then the deviance and Akaike information criterion (AIC) measures^22^ can be used for model fit and comparison.

## Illustrative Example: IVIG for Severe Sepsis and Septic Shock

### Background and CEA Model

Sepsis is a syndrome characterized by a systemic inflammatory response to infection that leads to rapid acute organ failure and potentially rapid decline to death. Severe sepsis (sepsis with acute organ dysfunction) represents approximately 31,000 patient episodes and 15,000 in-hospital deaths per year in the United Kingdom. Intravenous immunoglobulin (IVIG), a human blood product, has been proposed as an adjuvant therapy for severe sepsis, but evidence regarding the use of IVIG in severe sepsis is conflicting.^23^ We were commissioned to perform a systematic review, meta-analysis, CEA, and value-of-information analysis of IVIG for severe sepsis and septic shock, with an aim to assess the potential value and design of additional primary research.^14^ A full description of the studies and the data that were extracted is available in Tables 7 to 13 of this report.^14^ The primary effectiveness outcome from the RCT studies was all-cause mortality. The CEA model, comparing IVIG with standard care, consisted of a decision tree to model for short-term survival of a sepsis event and a Markov model for the mid- to long-term consequences of surviving sepsis. A meta-analysis of the RCT studies informed the relative effects of treatments on short-term mortality following a sepsis event. Other model inputs to the CEA model came from a variety of registry and cohort evidence sources (see report^14^ for full details). All programs and data available from NJW on request.

### Potential Covariates in the Meta-Analysis of RCT Studies

#### Treatment definitions

**Figure 1:**
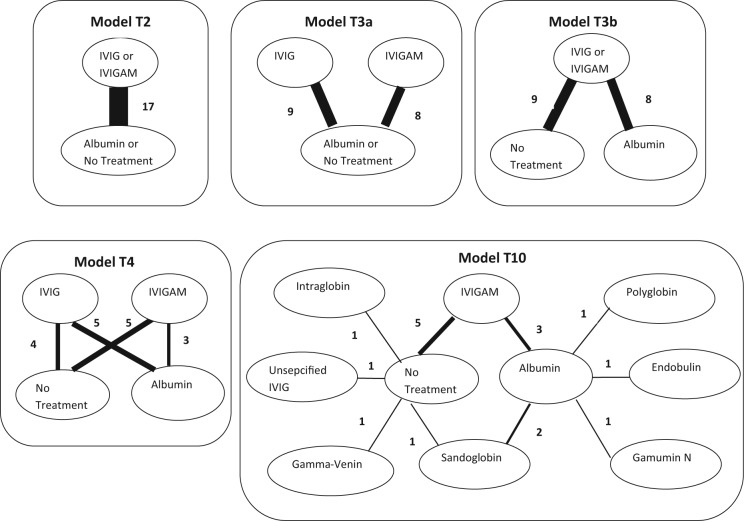
Network plots for each of the 5 treatment models considered. Treatments connected by a line indicate where randomized controlled trial (RCT) evidence is available, and the width of the line is proportional to the number of RCTs making that comparison. IVIG, intravenous immunoglobulin; IVIGAM, IgM-enriched IVIG.

In the 17 identified RCT studies, IVIG was either standard IVIG or IgM-enriched IVIG (IVIGAM) and differed in the duration of treatment (days), daily dose (g/kg^−1^/d^−1^), volume of fluid (mL/kg^−1^/d^−1^), and total dose (g/kg^−1^). Furthermore, there were several different formulations. All studies had two arms and used either albumin or no treatment (in addition to standard treatment) as control. For the different IVIG and control preparations, we considered 5 different possible treatment comparison models (numbered according to number of treatments), also displayed in Figure 1:

Model T2: (IVIG or IVIGAM) v. (albumin or no treatment)

Model T3a: IVIG v. IVIGAM v. (albumin or no treatment)

Model T3b: (IVIG or IVIGAM) v. albumin v. no treatment

Model T4: IVIG v. IVIGAM v. albumin v. no treatment

Model T10: sandoglobin v. intraglobulin v. gamma-venin v. polyglobin v. endobulin v. gamumin N v. IVIG unspecified v. IVIGAM v. albumin v. no treatment

We initially explored extending the range of treatment comparison models according to dosing regimen, but this did not always result in a connected network. Instead, we considered the attributes of the dosing regimen (average daily dose, volume, duration, and total dose) as arm-level covariates.

We consider the simplest treatment model T2 as the reference “no covariate” case (step 1) and explore each of the other more complex treatment effect models in turn alongside the univariable models for the other covariates (step 2). We have a preference for the simplest treatment model that achieves adequate fit (i.e., the most parsimonious).

#### Risk of bias

We also extracted the following risk of bias information: intention-to-treat analysis performed (yes/no), concealment of allocation to treatment (adequate/unclear/inadequate), blinding to treatment (adequate/unclear/inadequate), randomization procedure (adequate/unclear/inadequate), Jadad score^24^ (which is based on a composite score for adequacy of randomization [0–2 points], blinding [0–2 points], and presence or absence of attrition information [0–1 points], yielding a score from 0 to 5, where 5 represents the best quality score), publication date, and sample size (intervention arm), which were considered potential covariates.

#### Other study characteristics

We also considered as covariates whether the study reported that the trial was carried out in a critical care setting or not and follow-up period (weeks).

#### Patient characteristics

We included baseline risk (control arm log-odds of mortality) as a covariate. Although other covariates (such as scores for severity of illness and number and sites of organ failure) were considered possible effect modifiers, they were not reported in sufficient detail in enough studies to be explored.

### Model Selection

We used a Bayesian framework to fit the models and assess model fit using the posterior mean residual deviance, ${\bar{D}}_{\mathrm{res}}$, which in a adequately fitting model is expected to be approximately equal to the number of data points if we assume normality (here there are 2 × 17 = 34 data points for 17 two-arm studies). Models where ${\bar{D}}_{\mathrm{res}}$ is much larger than this display evidence of lack of fit. For model comparison, we also use the DIC, which provides a composite measure of model fit and model complexity, preferring models with lower DIC. Differences in ${\bar{D}}_{\mathrm{res}}$ and DIC of 3 or more are considered meaningful if we assume normality.^21^ We also inspect changes in the posterior mean of the between-studies standard deviation, $\bar{\tau }$, to observe how much heterogeneity has been explained by introducing covariates.

**Figure 2:**
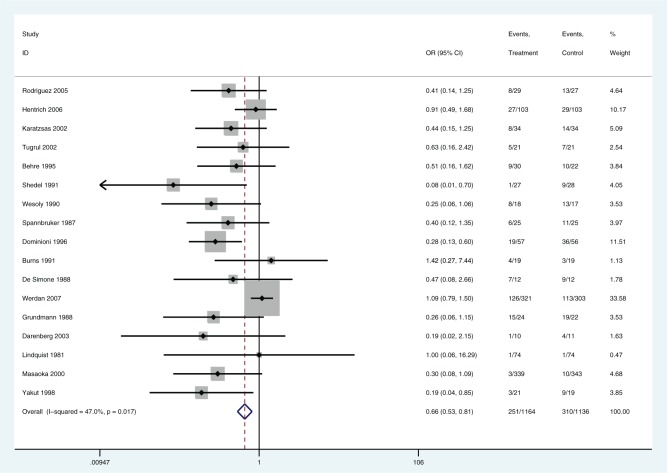
Forest plot for (IVIG or IVIGAM) v. (albumin or no treatment). Random effects model. IVIG, intravenous immunoglobulin; IVIGAM, IgM-enriched IVIG.

*Step 1: No covariates (model T2)*. The FE shows substantial lack of fit (${\bar{D}}_{\mathrm{res}}$ = 51.4 compared with 34 data points), whereas the random effects model fitted well (${\bar{D}}_{\mathrm{res}}$ = 30.9) (Table 1). This reflects the high degree of heterogeneity ($\bar{\tau }$ = 0.56 on a log-odds ratio scale; see also Figure 2).

**Table 1:** Model Selection Steps 1 and 2

Covariate Type	Covariate	FE		RE		
		${\bar{D}}_{\mathrm{res}}$	DIC	${\bar{D}}_{\mathrm{res}}$	DIC	τ
None	No covariates (Model T2)	51.4	188.2	**30.9**	**175.0**	0.56
Dosing regimen	** Duration of treatment (days)**	**37.1**	**175.0**	32.5	175.3	.38
	** Daily dose (g/kg^−1^/d^−1^)**	**36.9**	**174.6**	33.0	175.2	.36
	** Volume (mL/kg^−1^/d^−1^)**	**36.9**	**174.6**	33.2	175.3	.36
	Total dose (g/kg^−1^)	52.2	190.0			
Treatment model	T3a	50.1	187.9			
	** T3b**	**42.8**	**180.5**	**31.6**	**175.3**	**0.48**
	T4	43.6	182.3			
	T10	36.2	180.7			
Risk of bias	Intention-to-treat analysis (yes/no)	45.0	182.7			
	Adequacy of concealment of allocation to treatment	41.5	179.2			
	Adequacy of blinding to treatment	48.8	186.5			
	Adequacy of randomization procedure	45.2	182.9			
	** Jadad score**	**39.2**	**176.9**	**32.2**	**175.6**	**.45**
	** Publication date**	**35.9**	**173.7**	**33.1**	**174.8**	**.31**
	** $1/\sqrt{N}$ (*N* = number of patients randomized to IVIG arm)**	**36.6**	**174.4**	**32.7**	**174.6**	**.33**
Other	Critical care setting	51.6	189.4			
	Baseline risk (control arm log-odds of mortality)	53.0	190.8			
	Follow-up period (linear relationship)	46.5	184.3			
	Follow-up period (<4 or ≥4 weeks)	48.5	186.3			

Summaries of model fit beginning with the 2-treatment comparison model (model T2: (IVIG or IVIGAM) v. (albumin or no treatment)) and then adding single covariates individually. Different treatment models (in the absence of other covariates) are also compared. Summary statistics are the posterior mean residual deviance, ${\bar{D}}_{\mathrm{res}}$; the deviance information criterion, DIC; and the posterior mean of the between-trials standard deviation, τ. Key covariates that substantially improve model fit are highlighted in bold. For treatment models, where fit is comparable, the simplest model is highlighted. IVIG, intravenous immunoglobulin; IVIGAM, IgM-enriched IVIG; FE, fixed effects; RE, random effects.

*Step 2: Univariable models (single covariates).* The key covariates that appeared to explain some of the heterogeneity in the meta-analysis were dosing regimen covariates (duration of treatment, daily dose, and volume), treatment definition (T3b was the most parsimonious), and risk of bias covariates (Jadad score, publication date, and a measure of sample size: $1\;/\;\sqrt{N}$) (Table 1). Including any one of these key covariates resulted in reduction in ${\bar{D}}_{\mathrm{res}}$ and DIC in the FE model and a reduction in $\bar{\tau }$ in the RE model. Further improvement in model fit was obtained by treating each IVIG preparation as a separate treatment (T10); however, this model was more complex, and on the basis of DIC, model T3b was preferred. Follow-up period showed a mild effect, but this disappeared when any of the above key covariates were included (results omitted).

*Step 3: Multivariable models (combinations of key covariates).* There was no improvement in model fit from including all key dosing regimen covariates compared with including just one (Table 2). Similarly, for the “risk of bias” covariates, it was only considered necessary to include one of these covariates in further models (Table 2). Combining a dosing regimen covariate with a risk of bias covariate improved model fit and led to reductions in DIC (Table 2). This suggests that these two types of covariates measure different aspects of heterogeneity. Furthermore, for treatment model T3b, adding risk of bias covariates did not lead to much change in model fit, suggesting that the choice of control explains the same aspect of heterogeneity as the risk of bias covariates (Table 2). The FE models that give the lowest DIC are highlighted in bold in Table 2. The best-fitting FE model (and lowest DIC) was obtained for treatment model T3b with duration of treatment as a covariate (Table 2). This model fitted as well as the RE model with no covariates (Tables 1 and 2), suggesting that for this example, there are two dimensions of heterogeneity, one relating to the dosing regimen and the other to risk of bias.

**Table 2:** Model Selection Step 3

Covariates	Model T2		Model T3b	
	${\bar{D}}_{\mathrm{res}}$	DIC	${\bar{D}}_{\mathrm{res}}$	DIC
Duration of treatment (days)	37.1	175.0	**29.8**	**168.6**
Daily dose (g/kg^−1^/d^−1^)	36.9	174.6	37.4	176.2
Volume (mL/kg^−1^/d^−1^)	36.9	174.6	37.5	176.3
T3b			42.8	180.5
Jadad score	39.2	176.9	39.3	178.1
Publication date	35.9	173.7	36.4	175.2
$1/\sqrt{N}$ (*N* = number of patients randomized to IVIG arm)	36.6	174.4	36.1	174.9
Duration of treatment + daily dose + volume	34.3	173.6	**30.8**	**171.3**
Jadad score + publication date +$1/\sqrt{N}$	35.7	175.4	36.7	177.6
Duration of treatment + Jadad score	**33.4**	**172.3**	**30.7**	**170.5**
Duration of treatment + publication date	**31.4**	**170.2**	**30.1**	**169.8**
Duration of treatment +$1/\sqrt{N}$	**33.7**	**172.5**	**30.7**	**170.5**
Daily dose + Jadad score	37.4	176.2	38.0	177.7
Daily dose + publication date	34.6	173.3	35.6	175.4
Daily dose +$1/\sqrt{N}$	**32.2**	**171.0**	33.1	172.9
Volume + Jadad score	37.5	176.3	38.1	177.8
Volume + publication date	34.7	173.4	35.7	175.5
Volume +$1/\sqrt{N}$	**32.4**	**171.2**	33.3	173.1

Summaries of model fit for fixed effects models with combinations of the key covariates identified in steps 1 and 2 (Table 1). Summary statistics are the posterior mean residual deviance, ${\bar{D}}_{\mathrm{res}}$, and the deviance information criterion, DIC. Best-fitting models are highlighted in bold. IVIG, intravenous immunoglobulin.

*Step 4: Incorporation of expert opinion and sensitivity analyses.* We found that aspects of treatment regimen (duration of treatment, daily dose, volume) were associated with treatment effect. However, discussions with the expert advisory group for the project highlighted that there was no clinical mechanistic rationale why these aspects of treatment regimen would affect treatment effects across the ranges examined within the included studies. Because these aspects of treatment regimen were not compared *within* studies, there was the worry that the effects we had observed were subject to ecological bias, highlighting the lack of early phase studies for IVIG for severe sepsis and septic shock. In particular, studies with longer duration of treatment may reflect a healthier population that could be treated for longer. The expert advisory group agreed that risk of bias indicators were important covariates to include and that the choice of control could be a proxy for risk of bias because albumin resembles IVIG, indicating adequate blinding. We therefore report results from RE models with one key risk of bias covariate as a sensitivity analysis, in addition to results of the best-fitting model (FE model T3b with duration of treatment as a covariate). In these RE models, the heterogeneity that can be explained with the dosing regimen covariates was left unexplained, reflecting a belief that these covariates were a proxy for other, unmeasured, differences between the studies.

### Results from Meta-Analysis

Table 3 shows the results from the models identified in the model selection process. Results are reported for albumin as the comparator for treatment model T3b and for the following covariate values: duration = 3 days (in the absence of any other rationale, 3 days was the most commonly reported treatment duration in the included studies), Jadad score = 5 (least risk of bias), publication date = 2007 (most recent in included studies), sample size N→∞ (infinitely large study), and N=339 (the sample size for the IVIG arm of the largest of the included studies).

**Table 3:** Predicted Odds Ratio (95% Credible Intervals) for the Best-Fitting FE Model T3b with Duration of Treatment as a Covariate and for RE Models with a Key Risk of Bias Covariate (Reporting the Predictive Distribution, Option b)

Treatment Model	Covariate	Reports	Predicted OR (95% Credible Intervals)
FE T3b	Duration	IVIG v. albumin for duration = 3 days	0.75 (0.58, 0.96)
RE T3b	None	IVIG v. albumin predictive distribution (option b)	0.68 (0.16, 1.83)
RE T3b	None	IVIG v. albumin RE mean (option a)	0.60 (0.32, 0.95)
RE T2	Jadad	IVIG v. control predictive distribution (option b) for Jadad score = 5	0.83 (0.18, 2.13)
RE T2	Publication date	IVIG v. control predictive distribution (option b) for publication date = 2007	0.83 (0.24, 1.72)
RE T2	$1/\sqrt{N}$	IVIG v. control predictive distribution (option b) for N→∞	1.27 (0.25, 3.17)
RE T2	$1/\sqrt{N}$	IVIG v. control predictive distribution (option b) for N=339	0.92 (0.23, 2.10)

For illustration, results reporting the RE mean (option a) are presented for the RE model T3b. IVIG, intravenous immunoglobulin; FE, fixed effects; RE, random effects; OR, odds ratio.

For the RE models, there are a variety of options as to the relevant predicted treatment effect to report and use in CEA (options (a)–(e) above). No single study or subsets of studies were considered more relevant than the others to the decision setting, and so options (c) to (d) were not appropriate. There was no reason to expect that the heterogeneity between studies was inherent variability (e.g., between hospitals/centers) that would be experienced in a roll-out of the intervention, and so option (e) was not considered appropriate. As described above, the random effects mean, option (a), is unlikely to be a good summary in general, and so we report results from the predictive distribution, option (b), which reflects the additional uncertainty resulting from the unexplained heterogeneity. For comparison, we report both options (a) and (b) for the RE T3b model.

The results show that the uncertainty in the treatment effect estimate is smallest for the FE T3b model with duration as a covariate (Table 3). For the RE model T3b, the uncertainty in the treatment effect estimate is much wider for the predictive distribution compared with the RE mean summary (Table 3).

The results are highly sensitive to the choice of model, with predicted odds ratios ranging from 0.6 (IVIG beneficial), for an RE T3b model reporting the RE mean, to 1.27 (IVIG harmful), for the RE T2 model with N→∞ (albeit with very wide credible intervals). However, note that the predictions from this model involve an assumption that the relationship between treatment effect and sample size continues for sample sizes beyond the observed studies. The results with publication data and Jadad score are very similar, and since it is easier to justify making predictions for Jadad score = 5 than for a publication date of 2007, we use the model with Jadad score and not publication date in the CEA models.

### Results from the CEA and Value-of-Information Analysis

The results from the CEA were highly sensitive to the choice of model (Table 4), with the incremental cost-effectiveness ratio (ICER) ranging from £15,000 to IVIG being dominated. The optimal decision depends on the model used, especially for the £20,000 threshold. There is considerable uncertainty in the optimal treatment under all models, with the probability that IVIG is cost-effective at the £20,000 threshold ranging from 0.3 to 0.7, with the RE mean giving more certainty than the predictive distribution for model RE T3b (Table 4). The expected value of information for all parameters (EVPI) and for the relative effect parameters (EVPPI) indicate that, regardless of the model used, there is substantial potential value in new research, including a well-conducted RCT comparing IVIG with standard care (Table 4). However, the optimal design of such a study is highly sensitive to the model selected (Table 4), ranging from 800 to 1900 per arm.

**Table 4:** Results from the Cost-Effectiveness Model^14^ for the Best-Fitting FE Model T3b with Duration of Treatment as a Covariate and for RE Models with a Key Risk of Bias Covariate (Reporting the Predictive Distribution, Option b)

Model	ICER (IVIG v. Standard)	Prob(CE), £20,000 Threshold	Prob(CE), £30,000 Threshold	Pop. EVPI (10-y Time Horizon)	Pop. EVPPI (Relative Effects)	Optimal Sample Size, n*	ENBS at n*
FE T3b, duration = 3 days	£20,850	0.505	0.789	£393m	£174m	1900	£137m
RE T3b, IVIG v. albumin. predictive distribution (option b)	£16,177	0.597	0.707	£1017m	£718m	1200	£687m
RE T3b, IVIG v. albumin. RE mean (option a)	£15,488	0.721	0.871	£472m	£148m	1400	£116m
RE T2, Jadad = 5, predictive distribution (option b)	£19,968	0.502	0.611	£1367m	£1022m	800	£1011m
RE T2, $1/\sqrt{N}$(*N* = 339), predictive distribution (option b)	£28,520	0.404	0.514	£898m	£620m	900	£606m
RE T2, $1/\sqrt{N}$ (N→∞), predictive distribution (option b)	Dominated	0.275	0.348	£603m	£381m	800	£365m

For illustration, results reporting the random effects (RE) mean (option a) are presented for the RE model T3b. Results reported are the incremental cost-effectiveness ratio (ICER) for IVIG v. standard care; the probability that intravenous immunoglobulin (IVIG) is cost-effective, Prob(CE), at the £20,000 and £30,000 willingness-to-pay per quality-adjusted life year thresholds; the total population expected value of perfect information (EVPI); the population expected value of partial perfect information (EVPPI) for the relative treatment effect parameters; the optimal sample size of a new trial, n*, that maximizes the expected net benefit of sampling (ENBS); and the ENBS obtained at n*.

## Discussion

We have presented a structured model selection strategy to incorporate covariates in evidence synthesis of relative treatment effects and described different model summaries that can be used as inputs to CEA models, depending on how the heterogeneity in the evidence relates to the setting for the decision. Applying the methods to our illustrative example allowed us to identify models that eliminated the heterogeneity to an FE model through the inclusion of covariates, although we also presented results from RE models after incorporating expert opinion. There have been several previous meta-analyses conducted on IVIG for severe sepsis/septic shock,^25–30^ and conflicting conclusions have been drawn.^23^ Although all previous meta-analyses tested for heterogeneity, all (with the exception Turgeon et al.^27^) performed a fixed effects meta-analysis. Our findings from univariate analyses are in concordance with findings from previous meta-analyses, but our meta-analysis is the first to simultaneously allow for type of IVIG/IVIGAM, control treatment, study quality/publication bias, dosing regimen, and other potential covariates. Without exploring the model space fully and integrating expert input, the sensitivity of results to choice of model and interpretation of that model may be missed.

We found that clinical input is essential to obtain results that are interpretable and to help choose between competing models that fit equally well. In our illustrative example, the expert opinion was valuable to make us wary of overinterpretation of the best-fitting FE models, which included aspects of treatment regimen; present results from other models that do not include treatment regimen covariates; and help us understand the difference between treatment definitions, particularly the difference between the no treatment and albumin controls, and the link with risk of bias. Of course, expert opinion may also be subject to cognitive biases,^31,32^ such as confirmation bias, and in panels of experts the “bandwagon effect.”

We have proposed a particular model selection strategy, but other structured approaches could have been taken. To explore the relative performance of different selection strategies would require a detailed simulation study, which is an area for further research. When there is no other rationale to choose between models, sensitivity analysis is essential. Model averaging could be used to obtain a weighted analysis over the plausible model space.^33,34^ A fully Bayesian approach could also use expert opinion to obtain prior model weights for different covariate models. However, model averaging does not help with the interpretation and understanding of causes of heterogeneity and, as such, does not deliver any advantage over a simple RE model with no covariates.

Heterogeneity in treatment definition is common, and treatments are often grouped together (e.g., over dose or treatment class). Our approach can help determine whether grouping together is reasonable, again with clinical input. For IVIG, it was found to be reasonable to group treatments across different IVIG preparations. However, dosing regimen led to treatment effect modification and must be included as a covariate or as unexplained heterogeneity in an RE model. It was concluded that basic science was needed to better understand the mechanism of action of IVIG and to determine appropriate dosing regimens through dose-ranging studies.^14,35^

Risk of bias information is routinely collected, but we are unaware of other examples where bias adjustment has been used in CEA. We found that sample size was an important covariate. The natural way to adjust for bias due to small study effects is to predict the treatment effect as N→∞. Results were highly sensitive to this because if the relationship observed between treatment effect and sample size in the included studies were to continue as N→∞, then we predict that IVIG is harmful, albeit with high uncertainty. It is likely that IVIG is not considered likely to be harmful (otherwise the RCTs would not have been conducted), and so one possible solution would be to use an informative prior that gives relatively low weight to treatment effects that are harmful. This would need to be elicited from clinical experts.

We have used meta-regression methods to identify effect modifiers, but these methods suffer from low power to detect effects and are vulnerable to ecological bias.^6^ This is especially the case in the (common) situation where the spread of covariate values across studies is sparse (e.g., only a few studies where the covariate is absent). It was not possible to explore potential covariates where the data were sparse, although these may have been important treatment effect modifiers. Individual participant data (IPD) avoid many of these problems, although it may not be possible to obtain IPD from all (or any) of the included studies. There has been some recent work on methods for the combination of studies where there is IPD available for some but not all studies on a binary outcome.^36^ However, there is no real substitute for IPD when continuous patient-level characteristics are important effect modifiers. One important potential treatment effect modifier is baseline risk. We found that including baseline risk as a covariate in our example did not improve model fit (results omitted). Note that by modeling relative effects on the log-odds scale already imposes an interaction between baseline risk and relative effects on the absolute probability scale (with smaller absolute probability differences when baseline probability is close to 0 or 1), which perhaps explains why there was no added benefit of explicitly using baseline risk as a covariate. Careful attention needs to be given to the scale on which the model acts and an awareness of what this implies in terms of interactions on an absolute scale.

We have assumed that there is a single effectiveness parameter that inputs to the CEA model. In practice, there may be multiple outcomes measured in the studies included in the meta-analysis (e.g., all-cause mortality, risk of stroke and bleeds), and these may be correlated. Multivariate meta-regression models^37^ across outcomes will therefore be required. Methods for model selection with multiple outcomes and how the results from these models can be used in CEA is an area for further study.

We have focused on heterogeneity in relative treatment effects, but there may be heterogeneity in other inputs to a CEA model. In particular, heterogeneity in natural history parameters, such as baseline risk, is likely. Ideally, large cohort studies or registry data representative of the decision setting would be used to estimate baseline risk^38^ and relative effects from the evidence synthesis applied to the baseline risk to obtain absolute risk for use in the CEA model. Where subgroups according to baseline risk can be identified, then CEA results can be broken down by subgroup to give tailored treatment recommendations, or the subgroups can be averaged over to give a population average treatment recommendation, as described in equations (10) to (12). In the absence of relevant cohorts or registries, the “standard care” arms from the RCTs included in a meta-analysis or network meta-analysis that are considered representative of the decision setting may be used to estimate baseline risk. To avoid introducing bias in the relative treatment effects, a synthesis of the “standard care” arms should be done in a separate analysis from the synthesis of the relative treatment effects.^38^ All of the ideas presented in this article extend naturally to a synthesis of standard care arms to estimate baseline risk and also to any other model inputs that may exhibit heterogeneity.
